# Are Two Screws Enough for Fixation of Femoral Neck Fractures? A Case Series and Review of the Literature

**DOI:** 10.2174/1874325000701010004

**Published:** 2007-11-08

**Authors:** K.C Xarchas, C.D Staikos, S Pelekas, T Vogiatzaki, K.J Kazakos, D.A Verettas

**Affiliations:** Orthopaedic Department, Democritus University of Thrace, Alexandroupolis, Greece

**Keywords:** Femoral neck fractures, cannulated screws, fixation.

## Abstract

There is still a controversy in literature regarding the treatment of subcapital fractures of the hip with internal fixation. Different methods have been tested and studies such as in cadavers mainly prejudge the three cannulated screws application. We present a series of 20 patients in which percutaneous fixation with two parallel cannulated screws under specific technical conditions has led to an uneventful fracture union. No complications were observed at a one year follow-up. Reviewing the literature we found no previous clinical studies on the subject.

## INTRODUCTION

Subcapital hip fractures in younger patients, usually as a result of high-energy trauma, are considered to be rare and more frequent to men than women. The unusual fatigue fractures should also be taken into account, though such fractures are difficult to be diagnosed. Sufficient bone density is present, as opposed to elderly patients where such fractures are associated with osteopenia. Subcapital hip fractures in younger patients are generally treated with internal fixation rather than with primary hemiarthroplasty, which is generally reserved for older, low-demand patients. But what is the distinction of the age groups in order to determine the treatment method? Most of the authors reserve replacement for patients over 75 years of age. The decision should be certainly based on biological criteria rather than on the absolute age. Avascular necrosis can occur following this injury because of disruption of the femoral head blood supply. Therefore urgent fracture reduction is necessary to minimize the risk of avascular necrosis, and the golden standard is internal fixation through cannulated screws.

In this study we present our clinical experience with percutaneous application of two cannulated screws and review the relevant literature. By doing so we aim to contribute on the discussion whether two cannulated screws can provide sufficient fixation for this kind of fractures. We classify fractures according to the most commonly used radiological classification of Garden. Repeated studies of intra- and inter-observer variations have shown that this classification is only accurate for dividing fractures into those which are undisplaced (grade I and II) and displaced (grade III and IV). The Pauwels classification is based on the angle of the fracture line to the horizontal. The more vertical fractures will have a greater shearing stress across their surfaces and therefore a higher tendency for displacement under loading.It has been found to be of no clinical significance [1]. The recently devised AO classification of intracapsular fractures has been found to have no clinical relevance other than for the division into displaced and undisplaced. It was criticized as very complicated and therefore should not be used [2].

## MATERIALS AND METHODOLOGY

**Fig (1):**
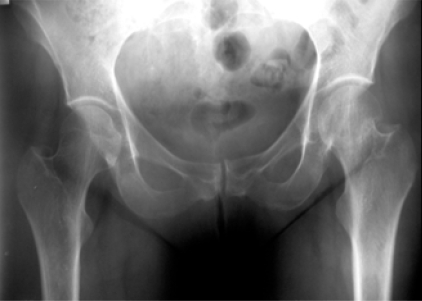
Garden I fracture. Preop.

**Fig (2):**
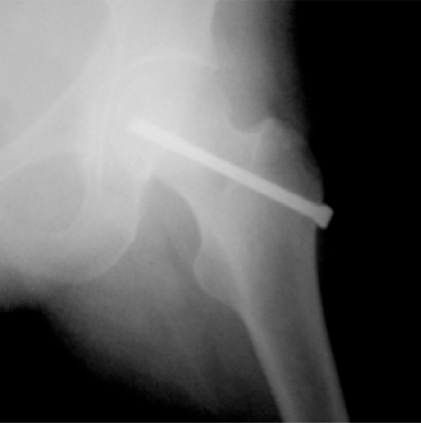
Garden I fracture. Postop AP.

**Fig (3):**
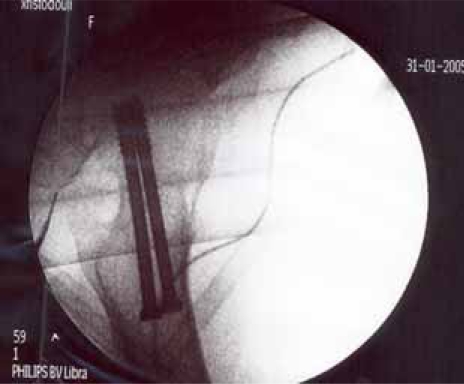
Garden I fracture. Postop Lat.

**Fig (4):**
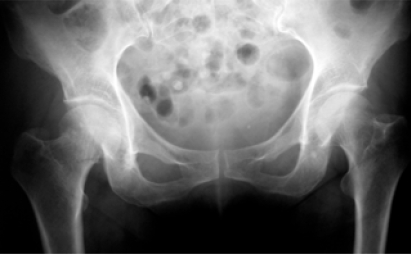
Garden II fracture. Preop.

**Fig (5):**
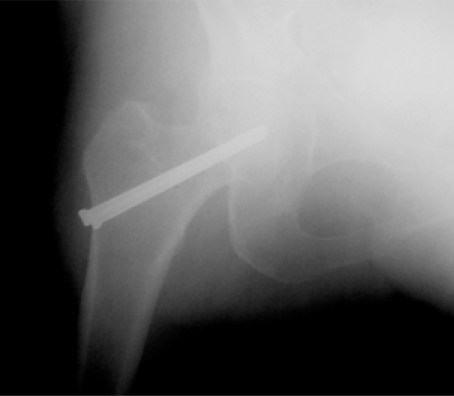
Garden II fracture. Postop AP.

**Fig (6):**
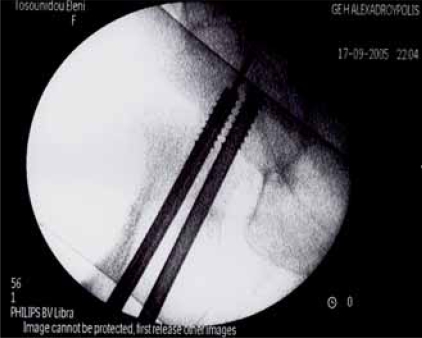
Garden II fracture. Postop Lat.

**Fig (7):**
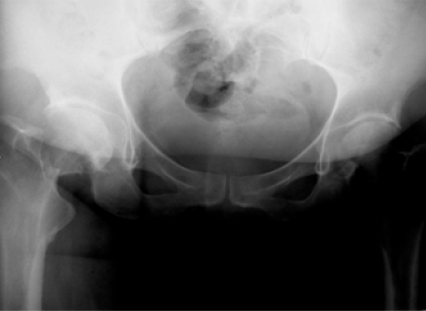
Garden III fracture. Preop.

**Fig (8):**
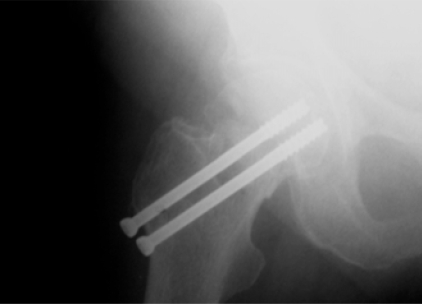
Garden III fracture. Postop AP.

**Fig (9):**
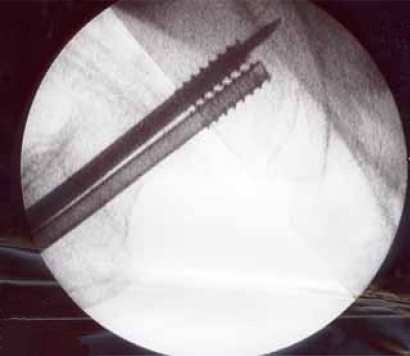
Garden III fracture. Postop Lat.

This study included 20 patients who had good general condition, no escorting diseases and were in excellent mental health. The mean age was 55 years old and there were six men and fourteen women. The younger patient was a 35 year old man and the oldest a 72 year old woman. The mechanism of injury was a simple fall in twelve cases, a fall from a height in six cases and a road traffic accident in two cases. There were fourteen right and six left hips. Regarding their occupation, there were ten pensioners, two manual workers, six involved in agricultural activities, one professional soldier and one inn-keeper. Seven patients suffered from Garden I type of fracture (Figs. **1, 2, 3**), other nine from Garden II (Figs. **4, 5, 6**), and four patients had Garden III subcapital fracture (Figs. **7, 8, 9**). Patients with Garden IV type of fracture were excluded from the trial as well as patients who had been treated with delayed fixation (more than twelve hours after the injury). We decided the exclusion of patients with a G IV fracture because in our experience such fractures most often require open reduction. In the entire series of twenty patients, fracture fixation was performed 6 to 8 hours after the injury. The research included patients that had been operated on during a time period of two years. One more year was allowed to complete one year follow up for all cases. During this period, there was one more patient with a G III fracture who met the criteria, but was not included in the study because of inadequate reduction, which led to failure of the fixation and a total hip replacement.

When necessary, manipulative closed reduction was carried out, using the fracture table and an x-ray image intensifier. Gentle longitudinal traction is first applied while screening in the anteroposterior view. After this the fracture is further reduced by internal rotation of the foot, while screening in the lateral view. This is the most important component of reduction of the fracture and full internal rotation is often necessary. The aim should be to achieve an anatomical or slight valgus position of the femoral head on the anteroposterior view. A varus position must be avoided. On the lateral radiograph, the femoral head, neck, trochanteric region and shaft should lie in a straight line. After reduction, true postero-anterior and lateral radiographs were retained for formal measurement according to the criteria of Garden: valgus 0-20^0^ to the postero-anterior aspect and ±20^0^ from the neutral position in the lateral view.

We applied two parallel cannulated screws percutaneously under fluoroscopic imaging up to the subchondral area (5mm. from joint line). Choosing the frontal plane, the lower screw was placed just superior to the calcar, with the tip in the subchondral bone close to the joint line. This gives a three-point fixation of the femoral head, calcar and the lateral cortical bone. The second screw was placed in parallel fashion centrally on the anteroposterior view. On the lateral radiograph, both screws were positioned in the middle and posterior part of the neck. When the sagittal plane was chosen, the first screw was positioned posteriorly and then a second parallel and in front of it with a tendency to spread them as possible, covering the neck in the lateral view. On the frontal plane screws were again introduced close to the calcar. The procedure was always carried out by the same surgeon. The cannulated screws used in our series (ASNIS-Stryker) have a shaft and wing diameter of 6.5 mm. The core diameter is 5.0mm. The average duration of the procedure was 15 min and the blood loss was less than 20ml. All patients were encouraged to mobilization within 24 hours and they were discharged two days after the operation. Shadow walking was immediately commenced and full weight bearing allowed on the sixth postoperative week.

## RESULTS

All patients were followed up at the outpatients department for one year at least. Monthly radiographic evaluation established fracture healing. All fractures united, and no patient underwent surgery for delayed union or nonunion. Avascular necrosis (assessed by clinical examination, xray control and MRI scan in only two suspicious cases) did not occur in any case and also no deaths, deep venous thromboses, or wound infections were noted. All patients had a decreased range of movement of the operated hip, especially in internal rotation but this was neither painful nor limiting their daily activities. Twelve patients returned to their previous occupations and the rest of them restricted their activities for subjective reasons. All patients were satisfied from the operation they had undergone.

## DISCUSSION- CONCLUSIONS

Cannulated screws are now universally used for the fixation of femoral neck fractures. They provide better fixation than pins which are known to have a significantly lower rate of nonunion and infection than the sliding screw-plate [3]. When directly compared to the screws, no advantage was shown for an implant with a side plate [4].

Screws should be applied in a parallel fashion [5] and when two screws are used, they should have a shaft diameter of at least 6.5 mm [6]. Positioning of a screw inferiorly and close to the calcar in the AP plane, offers cortical support to the screw and therefore better fixation. The same applies for posterior placement of a screw on the lateral view [7,8,9]. On the lateral view a reduced spread of the screws was associated with an increased risk of nonunion of the fracture [10]. These technical guidelines were strictly kept in our series forming the operative technique that has already been described.

Though there appears to be no clinical evidence indicating which design of screws is preferable, or if two, three or more screws are the best, biomechanic studies in cadavers [11,12] or using bone models [13] favor the application of three screws in a triangular fashion. On clinical grounds, Parker and Blundell [4] meta analyzed 25 randomized trials including 4,925 patients and found it impossible to determine the optimum number or type of screws.

The classic method of three cannulated screws is also used in our Department. In spite of its good results, longer operating time, increased irradiation and more demanding technique, were observed. Proper triangulation of the screws is extremely difficult to be achieved by closed means and may require the open technique to be used. Complications often related to this method including screw malpositioning , neck penetration and cut out were not met in our series.

**Fig (10):**
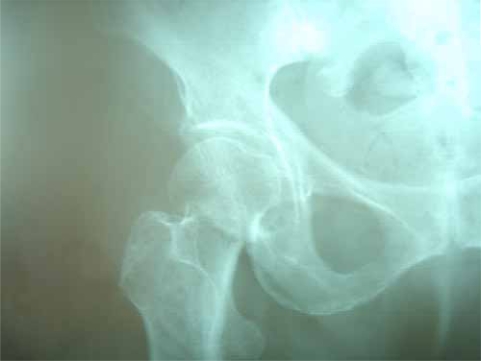
Fracture healing through impaction and backing of the screws. Preop

**Fig (11):**
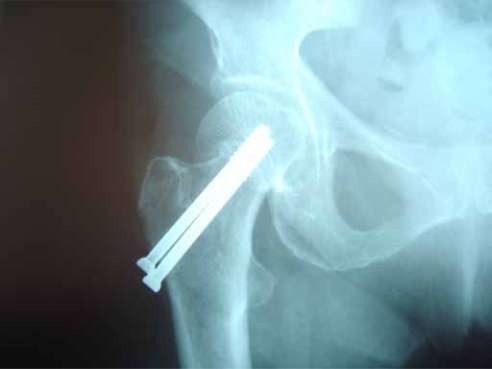
Fracture healing through impaction and backing of the screws. Postop at 3 months.

In our opinion, given the right conditions as reduction close to ideal, proper prompt fixation and early mobilization is probably more significant than querying two or three screws for fixation; we suppose the percutaneous application of two cannulated screws as a less harming method for bone and soft tissues, relates to quicker bone healing and minimal complications. If good reduction has been achieved, early weight bearing is possibly a contributing factor to fracture healing through impaction and backing of the screws (Figs. **10, 11**).

We do recognize that this is a case series with no control group. Our aim was to contribute on the discussion whether two cannulated screws can provide sufficient fixation for subcapital fractures of the neck of femur. It is certain that to give an answer on this controversial issue, larger and better designed studies will be required. Nevertheless, the effectiveness of the method in selected patients is very encouraging.
